# Evaluation of Misalignment Effect in Vehicle-to-Vehicle Visible Light Communications: Experimental Demonstration of a 75 Meters Link

**DOI:** 10.3390/s21113577

**Published:** 2021-05-21

**Authors:** Sebastian-Andrei Avătămăniței, Cătălin Beguni, Alin-Mihai Căilean, Mihai Dimian, Valentin Popa

**Affiliations:** 1 Integrated Center for Research, Development and Innovation in Advanced Materials, Nanotechnologies, and Distributed Systems for Fabrication and Control, Stefan cel Mare University of Suceava, 720229 Suceava, Romania; sebastian.avatamanitei@usm.ro (S.-A.A.); catalin.beguni@usm.ro (C.B.); dimian@usm.ro (M.D.); 2 Department of Computers, Electronics and Automation, Stefan cel Mare University of Suceava, 720229 Suceava, Romania; valentin@eed.usv.ro

**Keywords:** accident prevention, communication-based vehicle safety applications, inter-vehicle communications, long-range, V2V, vehicle misalignment, vehicle-to-vehicle communications, vehicle safety, vehicular communications, visible light communications

## Abstract

The use of visible light communications technology in communication-based vehicle applications is gaining more and more interest as the research community is constantly overcoming challenge after challenge. In this context, this article addresses the issues associated with the use of Visible Light Communications (VLC) technology in Vehicle-to-Vehicle (V2V) communications, while focusing on two crucial issues. On the one hand, it aims to investigate the achievable communication distance in V2V applications while addressing the least favorable case, namely the one when a standard vehicle rear lighting system is used as a VLC emitter. On the other hand, this article investigates another highly unfavorable use case scenario, i.e., the case when two vehicles are located on adjacent lanes, rather than on the same lane. In order to evaluate the compatibility of the VLC technology with the usage in inter-vehicle communication, a VLC prototype is intensively evaluated in outdoor conditions. The experimental results show a record V2V VLC distance of 75 m, while providing a Bit Error Ratio (BER) of 10^−7^–10^−6^. The results also show that the VLC technology is able to provide V2V connectivity even in a situation where the vehicles are located on adjacent lanes, without a major impact on the link performances. Nevertheless, this situation generates an initial no-coverage zone, which is determined by the VLC receiver reception angle, whereas in some cases, vehicle misalignment can generate a BER increase that can go up to two orders of magnitude.

## 1. Introduction

### 1.1. Visible Light Communications-Related Aspects

The attractiveness of optical wireless communication technologies has significantly increased in recent years with the introduction and growing popularity of the Visible Light Communications (VLC) concept [1]. VLC technology has grown from the status of an interesting idea to one of a technology that is almost ready for market deployment, bringing together an energy-efficient lighting technology and a health-safe data broadcasting solution. The wide distribution of LED light sources and the numerous benefits of VLC technology have attracted attention from numerous researchers and thus, a wide range of applications have been identified. Therefore, due to the unique properties of visible light, the VLC technology has been found to be suitable for underwater communications [2], whereas due to its lack of electromagnetic interferences, it is envisioned as a solution in potentially dangerous locations such as chemical plants, nuclear plants or in underground mines [3], where the usage of Radio-Frequency (RF)-based communications could be unsafe. The ubiquitous character of LED light sources also enables the VLC technology to be suitable in Internet of Things (IoT) applications [4,5]. Due to its ability to provide low latency, energy-efficient and cost-effective connections, the VLC technology has been found to be appropriate for industrial use, including in the Industry 4.0 domain [6].

Although numerous possible applications have been identified, the main usage of VLC technology is limited for now to high data rate indoor applications [7,8,9,10]. Indoor VLC systems have reached a rather high maturity level, with good performances, providing unprecedented data rates that currently go up to a few tens of gigabits per second (e.g., 20 Gb/s in indoor mobile settings [9], 15.73 Gb/s with off-the-shelf LEDs over a 1.6 m link [10],), whereas data rates that can go beyond 100 Gb/s are envisioned [11]. These unique perspectives, together with an inherent ability to provide small size isolated cells, allow the VLC technology to be considered a major candidate in Fifth Generation (5G) and Sixth Generation (6G) mobile and wireless networks [12,13,14]. It should be also mentioned that in addition to lighting and communication functions, the VLC technology has the potential to provide high accuracy indoor localization and positioning that reaches centimeter precisions [15,16], opening the door to a whole new range of applications [17], which in turn increases the need for a better mobility [18,19] and multi-user connections [20].

A particular use case for VLC technology is its use in vehicular applications. This concept envisions smart or autonomous vehicles that communicate with each other, or with intelligent traffic infrastructure, while using their already implemented LED lighting systems. Such applications have the potential to significantly contribute to a more efficient and safer transportation system. Nevertheless, unlike indoor applications, which imply rather stable conditions with a limited influence from parasitic light sources or from weather phenomena, vehicular VLC applications involve a variety of optical noise sources (i.e., natural and artificial), a greater degree of mobility, higher velocities and also a significant degree of unpredictability. In this context, different requirements and different results are expected. Indoor applications mainly require the ability to provide high-data rate short-range communications. On the other hand, vehicular applications require highly reliable, low latency, short-to-long-range communications. In such conditions, the data rate exigencies become less stringent, as the main purpose is to have an active link, no matter the circumstances.

### 1.2. Elements of Novelty in This Article

In light of the above, this article provides an analysis concerning the usage of the VLC technology in communication-based vehicle applications. Unlike most of the existing works on this technology, the present article addresses two of the most unfavorable use cases. First, instead of investigating the usage of a rather high power optical source, like a traffic light [21,22,23,24,25,26,27,28,29,30,31] or a vehicle headlamp system [32], this work investigates the capabilities of a VLC system based on LED rear lamps; this approach involves a significantly lower optical power. Secondly, this article also explores the effect of vehicle misalignment on the performances of a V2V VLC link. In this scope, a VLC test bench has been designed and implemented as a prototype, in order to experimentally investigate the possible communication distance of a V2V system, and also to use it in an experiment-centric approach in one of the unfavorable scenarios of vehicles misalignment, namely the one where the two vehicles are located on different lanes. To maintain a realistic approach and to ensure that the results are in accordance with the real-world conditions, the light distribution pattern of some existing commercial vehicles has been analyzed and compared to the light radiation pattern of the proposed VLC emitter. Next, the V2V VLC prototype has been used to recreate the scenario and to experimentally determine the performances of the V2V VLC link in two cases: one with the vehicles on the same lane and one with vehicles on adjacent lanes. In the end, the minimum communication distance, the achievable communication range and the Bit Error Ratio (BER) have been experimentally determined. The final results demonstrated a record V2V communication range of up to 75 m and BERs as low as 10^−7^ without the use of forward error correcting protocols. Thus, one can see that this article follows a highly realistic approach, analyzing less favorable situations, and more than this, it demonstrates improved performances with respect to the existing literature addressing vehicular VLC applications. This article continues the work started in [33], providing an in-depth theoretical and experimental analysis concerning the use of VLC technology in V2V applications. Unlike [33], which provided limited results valid only in night conditions, this article provides an exhaustive theoretical analysis, the intensive day testing demonstrating the effect of an adequate front-end block, together with improved results in terms of communication distance and BER performances. Furthermore, this article also provides a comparison between the results achieved in night conditions and in day conditions along with a comprehensive discussion concerning the future of VLC in automotive applications.

The rest of this article is organized as follows. Section 2 provides a summary concerning the progress of vehicular VLC applications. Section 3 presents an analysis concerning the influence of the incidence angle on the quality of the vehicle-to-vehicle visible light communications link. Section 4 describes the experimental setup for the vehicle rear lighting system irradiance determination and presents the results showing the irradiance pattern of two commercial vehicles. Section 5 provides the description of the VLC prototype that was used for the experimental tests, whereas Section 6 describes the outdoor testing procedure. Section 7 delivers the experimental results, followed by Section 8, which provides a discussion on the results and emphasizes the contributions of this work. Finally, Section 9 ends this article by summarizing the conclusions of this work.

## 2. Vehicular Visible Light Communications and Related Work

In the early 2000s, VLC technology emerged as a technology initially envisioned for automotive applications [34]. After a promising beginning, there was almost a decade when the development of vehicular VLC prototypes was rather neglected, with very few research groups investigating the topic. In the 2010s, the development of vehicular VLC systems gained momentum [35,36,37] and so the performances in this area have gradually improved. In terms of light-sensing, the design of automotive VLC receivers followed two paths, one based on photodiodes, especially PIN photodiodes, and the other one based on camera systems, each one with its advantages and drawbacks, as underlined in [34] and [38].

In recent times, the performances of vehicular VLC prototypes significantly improved in terms of communication distance, and data rates [34,39]. Furthermore, in terms of reliability, it has been demonstrated that using proper optical systems [30,40,41] and signal processing techniques, automotive VLC prototypes can maintain the connectivity link in fog conditions [42,43], in snowfall conditions [23,24] or in direct exposure to natural and artificial light sources [25,26,27], while providing extremely low latencies that go down to a few milliseconds [23,28]. In terms of coverage, the communication distance of existing prototypes varies from an average of 50 m [23,26,27,28,29,44] to distances of around 100 m [31,45,46], where the reliability of the link is determined by the lighting conditions and by the data rate. Automotive VLC systems are able to provide relatively high data rates over short distances, such as 427.5 Mb/s over 1.2 m, as seen in [32], but rarely provide more than a few tens of megabits per second rates over useful distances. Megabits per second data rates over a few tens of meter distances have been demonstrated in [45,46]. On average, such systems provide reliable middle-range links while supporting tens to hundreds of kilobits per second data rates [23,26,27,28,29,44]. Since the performances of automotive VLC systems are influenced by a wide range of factors, an adequate solution would be to use environment or context-adaptive VLC architectures [47,48], which evaluate the existing circumstances and determine an optimal trade-off for each communication parameter. Although remarkable progress has been made in addressing the main issues affecting the automotive VLC system performances, most of these challenges remain active topics of interest, as their further improvement is still being researched [34].

Therefore, in order to be suitable for communication-based vehicle applications or for autonomous vehicles, the reliability, the communication distance, and the data rates are still on the list, while an intrinsic challenge begins to be re-exposed. On this topic, in order to have an active link between the VLC emitter and the VLC receiver, a mandatory Line-of-Sight (LoS) condition is required. Nevertheless, in mobile scenarios associated with the vehicular environment, there could be certain situations when the mandatory LoS condition is not always satisfied. Previous research based on the analysis of the VLC channel has indicated that the VLC technology could be used in platooning applications [49,50]. Moreover, other studies showed that the VLC technology, in a hybrid architecture, can significantly enhance the resilience to malicious attacks and the reliability of the link for 802.11p 5.9 GHz inter-vehicle communications [51,52,53]. This observation further highlights the important role of VLC in automotive applications, as it has a high complementarity with the RF-based Wireless Access in Vehicular Environments (WAVE) solutions. In such hybrid scenarios, the VLC technology is suitable for short to middle-range direct Vehicle-to-Vehicle (V2V) connections, whereas RF-based solutions are more adequate for long-range non-LoS situations and for broadcasting messages that are relevant for the entire chain of vehicles. On the other hand, for messages that are only relevant in a relatively small area, the VLC solution is more adequate, as it contributes to spectrum offloading due to the inherent properties of light, which in this case provide a spatial isolation between neighboring communication channels. Thus, this feature enables an efficient location-based data distribution.

One can see that the use of VLC in vehicular applications can bring some benefits, making this technology a suitable candidate in providing inter-vehicle connectivity in autonomous vehicle applications [54,55,56]. However, due to the mandatory LoS conditions imposed by the intrinsic features of this technology, the reliability of the link connection is hard to be maintained, as there are many variables that influence it in V2V applications. To overcome this challenge, different approaches have been made for various driving scenarios. For instance, the authors of [57] propose the usage of a lane-centering (LC) technique that maintains the vehicles aligned in the middle of the lane, helping in this way to maintain the connectivity of the V2V VLC link. The problem of vehicle misalignment was also addressed in [58]. In this case, the authors focused on the case when the misalignment is caused by the vehicle trajectory at curved sections of the road. The analytical evaluation based on simulations showed that in such conditions, the connectivity of the link is affected. Therefore, in the case of a VLC emitter with a half value angle of ±15° and a receiver with a FOV of ±30°, the connectivity is lost at angles above 17°. To overcome this issue, the authors propose the usage of relay-assisted communications supported by an intermediate vehicle. Although the analysis provided by [58] is interesting, it should be mentioned that the analyzed scenario is based on a Lambertian radiation pattern and not on a real vehicle light distribution pattern, which can have a great impact on the final result, as demonstrated in [59] and in [60].

The VLC technology should provide reliable communications while maintaining the lighting device’s primary purpose. Thus, the light radiation pattern and the output optical power must be in compliance with the regulations, even if their adjustment would significantly optimize the SNR and the communication coverage. On the other hand, the performances of automotive VLC receivers can be significantly improved by narrowing the FOV. A narrow FOV limits the amount of optical noise (i.e., from sun and/or from artificial light sources with no data transmission capabilities) that reaches the optical detector, being one of the simplest solutions to enhance the SNR and to prevent the photosensitive element saturation. Nevertheless, narrowing the FOV also limits the mobility of the system, as the useful light can be also obstructed from reaching the VLC receiver. Therefore, the problem of maintaining the direct LoS between vehicles is very complex and challenging and requires further investigation.

## 3. Analysis Concerning the Influence of the Incidence Angle on the Quality of the Vehicle-to-Vehicle Visible Light Communications Link

**Figure 1:**
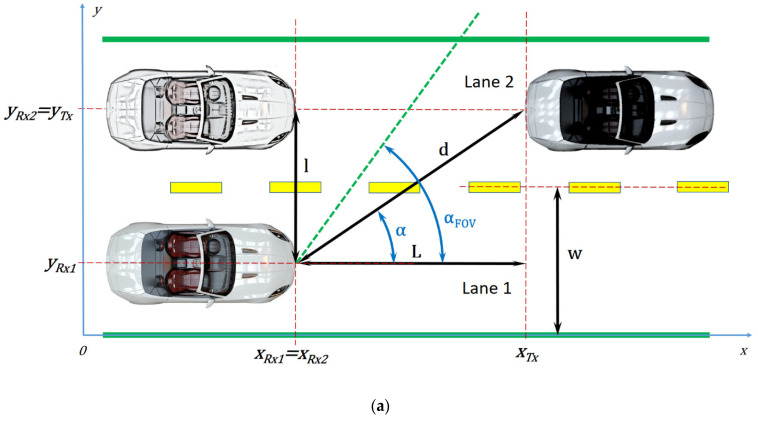

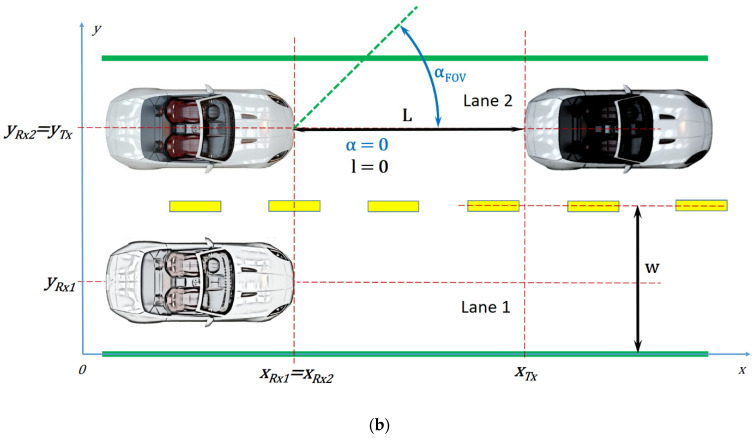
V2V communication scenarios: (**a**) Case one, with the communication established between two vehicles on adjacent lanes; and (**b**) case two, with the communication established between two vehicles on the same lane.

In order to highlight the communication differences between two vehicles on the same lane and two vehicles on adjacent lanes, the simulation scenario considers a straight road with two lanes traveling in the same direction. This test road is considered to have a 3.50 m lane width *w* (motorways have at least two through-traffic lanes in each direction, with a typical width between 3.50 m and 3.75 m each [61]) and the assumption made is that the vehicles will occupy the center of its corresponded lanes, at 1.75 m and, respectively, at 5.25 m projected on the *y*-axis. Another assumption is that the photodetector is centered along the longitudinal axis of the receiving car. The transmitting vehicle and the receiving vehicle are simulated with two test benches, the former equipped with two off-the-shelf vehicle rear lights, and the latter with a PIN photodiode-based PDA100A optical detector, situated at the same height *h* as the transmitting lamps, which can be fitted with an optical collimator based on a 2-inch diameter lens, having a FOV of ±20°. In order to make the measurements, it is easier to consider two cases: one where the transmitting vehicle is on one of the lanes (e.g., the second one) and the receiving vehicle is on the adjacent lane (e.g., the first one) as in Figure 1a, and the other where both cars are on the same lane (e.g., the second lane), as in Figure 1b. It can be noted that the latter situation is a particular case of the former, where the angle of incidence α has 0°, and the lateral distance *l* is zero.

**Figure 2:**
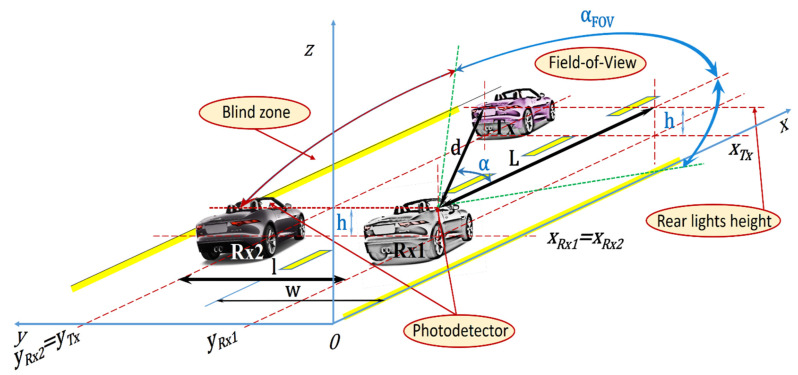
Vehicle configuration for analytical study.

The simulation scenario is further divided in measurement steps where the longitudinal distance *L* between the vehicles is gradually increased from 1 m to 75 m, and the measurements are made alternatively for those two cases already mentioned. In order to make an analytical analysis, only the first case will be considered, as in Figure 2, the second case being easy to calculate, as already mentioned, for α=0° and l=0.

The receiving vehicle will occupy alternatively the positions Rx1 and Rx2, while the transmitting vehicle will occupy the position Tx, at a variable distance *L* from the receiving car. The position coordinates will be $x_{Rx1}$, $x_{Rx2}$, $x_{Tx}$, $y_{Rx1}$, $y_{Rx2}$, and $y_{Tx}$.

From Figure 2, the following system of equations can be determined:

(1) $$x_{Rx1}=x_{Rx2}=x_{Rx},$$

(2) $$x_{Rx}+L=x_{Tx},$$

(3) $$y_{Rx2}=y_{Tx},$$

(4) $$y_{Rx1}+l=y_{Rx2},$$

(5) $$\tan\alpha =\frac{l}{L},$$

(6) $$d=\frac{L}{\cos\alpha },$$

where *d* is the distance between cars.

Taking into account that the measurements will be made with and without the optical collimator, when the cars are on adjacent lanes, the photodetector will have a blind zone until $L=l/\tan\alpha_{FOV}$ for $\alpha \geq \alpha_{FOV}$, meaning around 9 m and 3 m, respectively.

An optical wireless channel with intensity modulation/direct detection (IM/DD) can be modeled as a baseband linear system that respects the following equation [62]:

(7) y(t)=R·x(t)⊗h(t)+n(t),

where *x*(*t*) is the instantaneous optical power of the emitter, *y*(*t*) is the photocurrent at the receiver level, *R* is the receiver responsivity, *h*(*t*) is the impulse response, *n*(*t*) is the signal-independent noise, and the “⊗” symbol denotes convolution. Being instantaneous optical power, *x(t)* is non-negative, and the average transmitted power respects the equation:

(8) $$P_{t}=\underset{T\to \infty }{\lim}\frac{1}{2T}\overset{T}{\underset{-T}{\int }}x\left(t\right)dt$$

This means that the average received power can be written as:

(9) $$P_{r}=H\left(0\right)P_{t},$$

where the channel DC-gain is:

(10) $$H\left(0\right)=\int_{-\infty }^{\infty }h\left(t\right)dt.$$

For $0\leq \alpha \leq \alpha_{FOV}$, in order to estimate the influence of the angle of incidence over the VLC link performance, the BER will be used as a term of comparison. First, the channel DC-gain will be determined [62]:

(11) $$H\left(0\right)=\frac{A}{d^{\gamma }}R_{0}\left(\alpha \right)T_{s}\left(\alpha \right)g\left(\alpha \right)\cos\alpha \;,$$

where *A* is the active area of the photodetector, $R_{0}\left(\alpha \right)$ is the radiant intensity of the transmitter, *g*(*α*) is the collimator’s gain, $T_{s}\left(\alpha \right)$ is the transmission factor of the optical filter, and γ is the path loss exponent. Considering a generalized Lambertian radiant intensity, the following relation can be written:

(12) $$R_{0}\left(\alpha \right)=[\left(m+1\right)/2\pi ]\cos^{m}\alpha \;$$

with order *m* being dependent on the transmitter’s semi-angle at half power Φ12:

(13) m=−ln2ln(cosΦ12), 

The collimator’s gain is dependent on the FOV and on the refractive index *n* of the collimator:

(14) $$g\left(\alpha \right)=\frac{n^{2}}{\sin^{2}\alpha_{FOV}}.$$

If the optical filter can be approximated with an omnidirectional filter, the signal transmission $T_{s}\left(\alpha \right)$ will have a relatively constant value, $T_{s}$. If the average transmitted power is $P_{t}$, the average received power *P_r_* is given by Equation (9), and the signal-to-noise ratio (SNR) can be expressed as:

(15) $$SNR\left(\alpha \right)=\frac{{\left(RP_{r}\right)}^{2}}{\sigma^{2}},$$

where *σ* is the noise variance, and *R* is the responsivity in A/W.

After all the calculations involved, the SNR function will be:

(16) $$SNR\left(\alpha \right)={\left[\frac{RP_{t}AT_{s}n^{2}\left(m+1\right)}{2\pi \sigma L^{\gamma }\sin^{2}\alpha_{FOV}}\right]}^{2}\cos^{2\left(m+\gamma +1\right)}\alpha .$$

For α=0 the *SNR* will be:

(17) $$SNR\left(0\right)={\left[\frac{RP_{t}AT_{s}n^{2}\left(m+1\right)}{2\pi \sigma L^{\gamma }\sin^{2}\alpha_{FOV}}\right]}^{2},$$

so:

(18) $$SNR\left(\alpha \right)=SNR\left(0\right)\cos^{2\left(m+\gamma +1\right)}\alpha .$$

Using an on-off keying (OOK) modulation with Manchester code, the BER can be expressed as:

(19) $$BER\left(\alpha \right)=Q\left(\sqrt{SNR\left(\alpha \right)}\right)=Q\left(\cos^{m+\gamma +1}\alpha \sqrt{SNR\left(0\right)}\right),$$

where the *Q*-function is:

(20) $$Q\left(x\right)=\frac{1}{\sqrt{2\pi }}\int_{x}^{\infty }e^{-y^{2}/2}dy.$$

As a result:

(21) $$BER\left(\alpha \right)=\frac{1}{\sqrt{2\pi }}\int_{\cos^{m+\gamma +1}\alpha \sqrt{SNR\left(0\right)}}^{\infty }e^{-y^{2}/2}dy=\frac{1}{\sqrt{2\pi }}\int_{\sqrt{SNR\left(0\right)}}^{\infty }e^{-y^{2}/2}dy+\frac{1}{\sqrt{2\pi }}\int_{\cos^{m+\gamma +1}\alpha \sqrt{SNR\left(0\right)}}^{\sqrt{SNR\left(0\right)}}e^{-y^{2}/2}dy,$$

so:

(22) $$BER\left(\alpha \right)=BER\left(0\right)+\frac{1}{\sqrt{2\pi }}\int_{\cos^{m+\gamma +1}\alpha \sqrt{SNR\left(0\right)}}^{\sqrt{SNR\left(0\right)}}e^{-\frac{y^{2}}{2}}dy.$$

**Figure 3:**
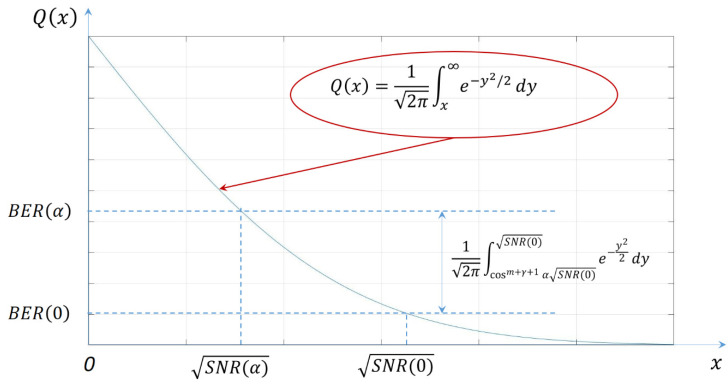
Bit Error Rate analysis.

The Q-function shown in Figure 3 shows that once the incidence angle α is increased, starting from 0°, then the SNR value is decreased and, consequently, the bit-error rate is affected, so one could expect to see this BER evolution with the experimental setup.

## 4. Experimental Determination of the Light Radiation Pattern of Commercial Vehicle Rear Lights

### 4.1. Measurement Settings

The performances of a V2V VLC link are significantly influenced by the amount of light-containing data that reaches the surface of the VLC receiver’s photosensitive element. In addition to the influence of the VLC channel [62,63,64], this amount of light is determined by the distance and by the angle between the VLC receiver’s axis and the direction of communication formed with the VLC emitter. Therefore, prior to proceeding to the V2V experimental examination, this section provides an analysis focused on the light radiation pattern experimentally determined for two commercial backlights. This intermediate step is highly important in order to correlate the parameters of automotive VLC prototypes with the premises imposed by the real-world scenario [59,60]. Thus, based on an objective approach, the performances of such VLC prototypes will be highly similar to the ones achieved by vehicle-embedded VLC systems.

**Figure 4:**
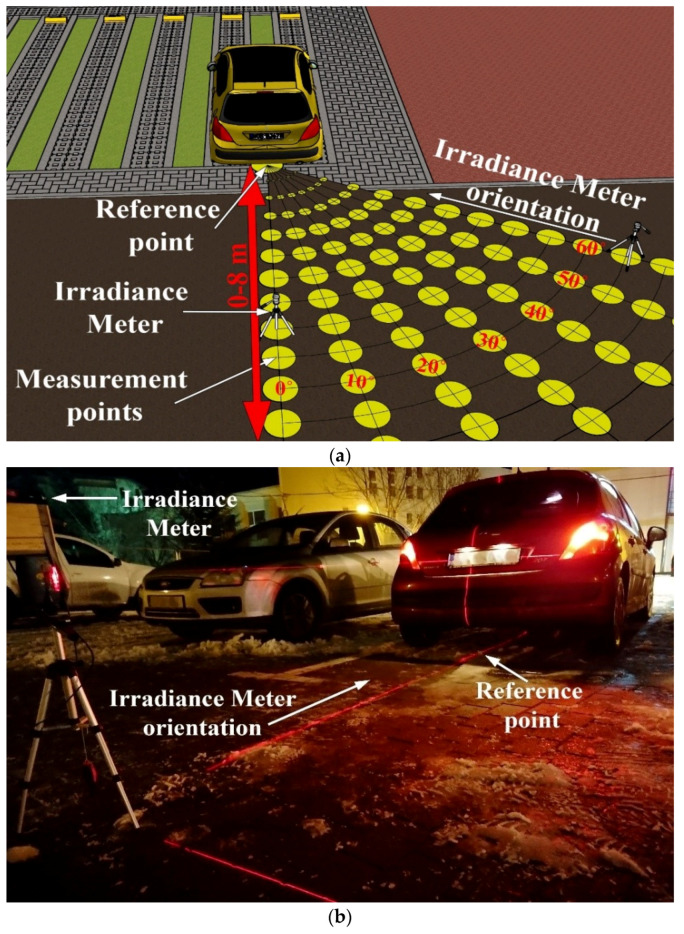
Vehicle light irradiance experimental measurement procedure: (**a**) envisioned testing scenario; (**b**) experimental testing setup.

In the first case, the light radiation pattern was determined for a vehicle with an LED-based lighting system. In the second case, the irradiance pattern of a twelve-year-old vehicle with a classical lighting system based on incandescent lights has been determined. Two different vehicles have been chosen in order to have a broader view concerning the light distribution pattern of different rear lighting systems available on commercial vehicles. The irradiance distribution patterns for the two vehicles have been determined both for taillights and for brake lights. These measurements have been performed with the help of a high precision irradiance meter with a resolution of 0.01 µW/cm^2^ (i.e., Delta Ohm 2302 using a LP 471 RAD probe). As illustrated in Figure 4a, the irradiance determination began at 0° and continued up to 60°, with measurements at every 10°. During these measurements, the irradiance meter was orientated toward the reference point, which is at the center of the back of the car. In terms of distances, the irradiance was determined from 1 to 8 m in steps of 50 cm. The experimental irradiance measurement procedure is illustrated in Figure 4a, whereas the setup for this procedure is shown in Figure 4b.

### 4.2. Discussion on Measurement Results

In line with what could be expected, these experimental determinations have shown that the vehicles’ rear lighting systems do not follow a Lambertian light distribution pattern. The explanation for this is that usually, the vehicle lights have irregular reflective surfaces behind each individual LED and/or small lenses in front of them, to keep the radiation pattern in line with the safety regulations.

**Figure 5:**
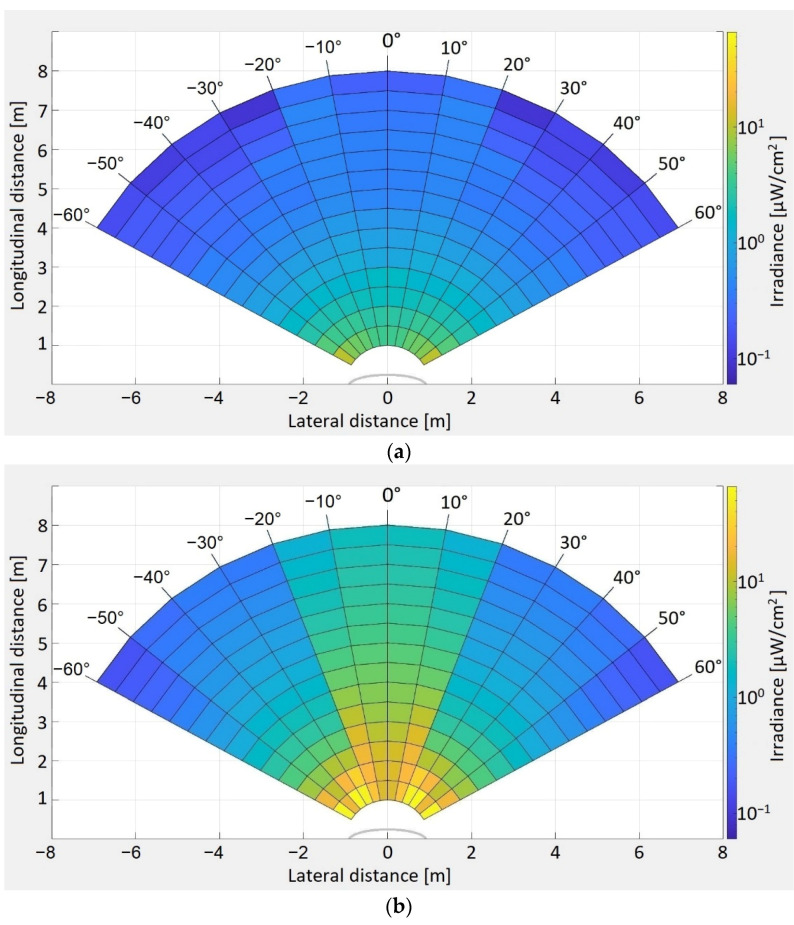

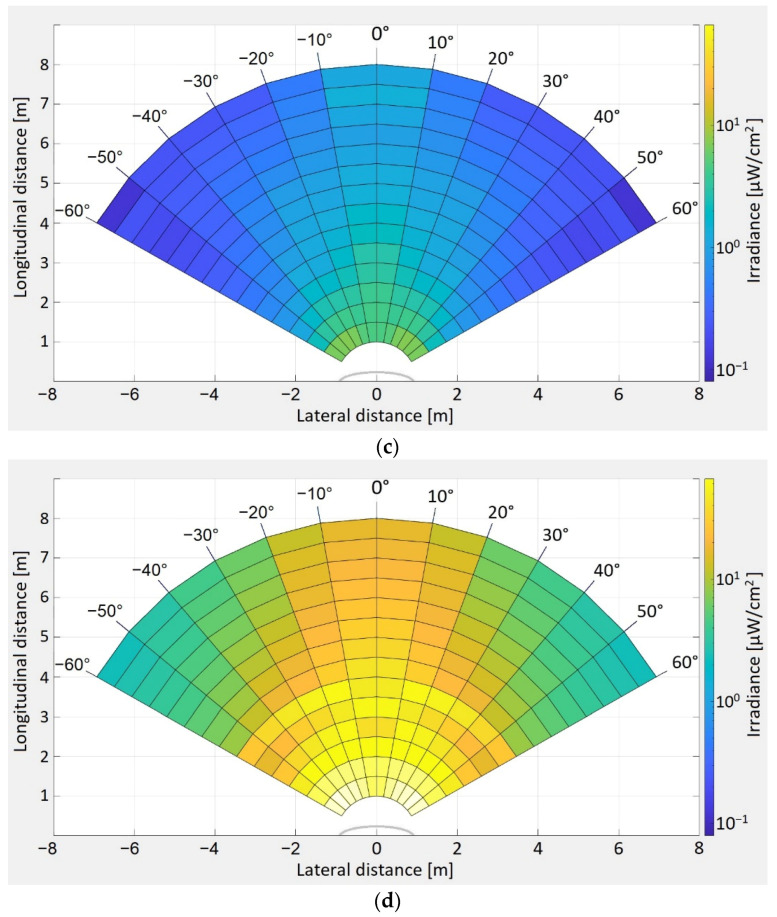
(**a**) Taillight irradiance distribution pattern for a vehicle with an LED-based lighting system; (**b**) stoplight irradiance distribution pattern for vehicle with an LED-based lighting system; (**c**) taillight irradiance distribution pattern for vehicle with incandescent based lighting system; and (**d**) stoplight irradiance distribution pattern for vehicle with incandescent based lighting system.

The experimental determinations showing the irradiance distribution patterns for the two vehicles are illustrated in Figure 5. The measured values for irradiance range as follows: from 10.46 µW/cm^2^ to 0.06 µW/cm^2^ for taillights with LEDs, from 66.4 µW/cm^2^ to 0.18 µW/cm^2^ for stoplights with LEDs, from 10.47 µW/cm^2^ to 0.08 µW/cm^2^ for taillights with incandescent bulbs, and from 317.5 µW/cm^2^ to 2.17 µW/cm^2^ for stoplights with incandescent bulbs. As expected, the light irradiation pattern has the highest values right behind the vehicle, and gradually decreases as the distance increases or as the measurement angle increases. The irradiance values also show that the brake lights have a higher intensity compared to the taillights, indicating their suitability for use in Emergency Electronic Brake Light (EEBL) applications, since the higher irradiance will provide an increased communication distance and enhanced resilience. Additionally, these determinations indicate that the irradiance of LED-based vehicle stop lights is significantly lower than the one of incandescent rear lighting systems. This fact indicates that the optical power of LED lighting systems could be further increased, contributing to an improved perception and to enhanced VLC performances.

Because these measurements were made while orientating the irradiance meter toward the center of the vehicles, in the real-world scenario when two vehicles are situated on different lanes while having the same direction, the quantity of light that reaches the photosensitive element is influenced by the incidence angle, as expressed in Equation (11). Thus, in such cases, the light that is actually received is lower than the values determined and illustrated in Figure 5. However, as the inter-vehicle distance increases, the incidence angle decreases, reducing the effect of vehicle misalignment.

## 5. Description of the Vehicle-to-Vehicle Visible Light Communications Prototype

**Figure 6:**
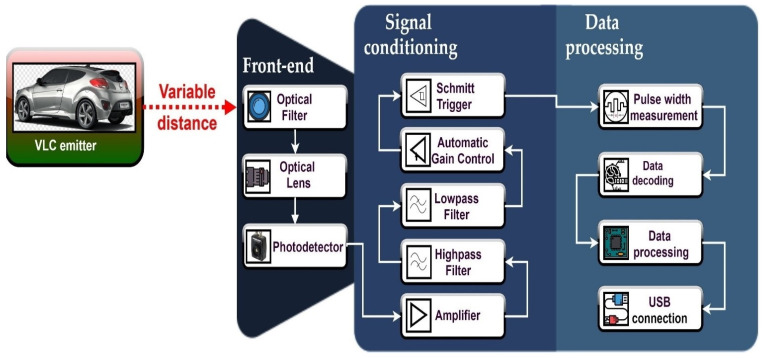
Schematic representation of the VLC system.

As illustrated in Figure 6, the V2V VLC prototype consists of a VLC emitter and a VLC receiver separated by the VLC channel. The VLC emitter mainly consists of a 180 MHz microcontroller board, an LED driver and an LED-based vehicle lighting system. The 180 MHz microcontroller is the main part of the VLC emitter. The microcontroller processes the data to send, transforms it into a binary string, encodes the data, creates the data frame and modulates it. In this case, the VLC prototype is able to work with Direct Sequence Spread Spectrum (DSSS) and On-Off-Keying (OOK) modulations, Sequence Inverse Keying (SIK) [21], Manchester and Miller [65] coding techniques and variable data rates between 3 and 200 kb/s. The data frame begins with a synchronization header that informs the VLC receiver that a new data frame will begin, continues with a physical header that provides the VLC receiver with information concerning the selected modulation, coding technique, data rate, and message length, and ends-up with a variable length data field. After the data frame is constructed, the information is modulated and the signal is applied to the LED driver, which in turn commands the LED vehicle rear lights, the data to send being transformed into a modulated light beam. The VLC emitter system is developed based on the characteristics of the LED vehicle backlights, in order to have an average transmitted optical power similar to the one of existing commercial vehicles.

The VLC receiver is the main component of a VLC system, as its design is the one that has the strongest impact on the VLC system performances. The VLC receiver consists of three main blocks. The front-end contains an optical collecting system that determines the VLC receiver FOV and its optical collecting area. As already mentioned in Section 2, the FOV strongly influences the VLC receiver’s SNR and its ability to work in mobile conditions. In order to evaluate the effect of the VLC receiver FOV on the system’s performances, two different FOVs have been used during the tests. Therefore, in the first case, the VLC receiver has been evaluated without an optical collecting system that limits its reception area, having this way a wide ±53° FOV. Next, in order to evaluate the effect of a narrower FOV, a 2-inch optical lens with a ±20° FOV has been used. The wide angle FOV should enable a wider communication area at the cost of a lower SNR, whereas the ±20° FOV should facilitate a SNR enhancement, contributing to a longer communication distance. The front-end also englobes an optical filter that eliminates part of the unwanted signals. In this case, an 80 nm band-pass optical filter with a center wavelength of 645 nm has been used. This optical filter is adequate, as the LED vehicle rear lights have only the red color lamps wired for communications. The selected optical filter eliminates up to 80% of the ambient optical noise. After that, the optical signal reaches the PDA100A PIN photodiode-based optical detector. The optical detector generates an electrical current, which is directly dependent on the power of the incident light. The internal transimpedance circuit transforms the electrical current into a voltage that will be further processed in order to extract the information.

The next stage of the VLC receiver is responsible for signal conditioning. At this stage, the low-amplitude signal is amplified, and then is passed through a band-pass filter. In order to mitigate the effect of artificial light sources, which mainly introduce a strong 100 Hz sinusoidal component, the band-pass filter has a high-pass cutoff frequency of 1 kHz, whereas in order to reduce the effect of high frequency noise sources (i.e., shot noise and thermal noise), the low-pass filter has an adjustable cut-off frequency, which for these tests was settled at 200 kHz. It should be mentioned here that the cut-off frequency of the low-pass filter is established based on a Power Spectral Density (PSD) analysis that is determined for each coding technique and for each data rate [29,65]. After the band-pass filter, the signal passes through an Automatic Gain Control (AGC) circuit, that requires input signals with a minimum amplitude of 200 mV, in order to provide an adequate amplitude signal for the Schmitt trigger circuit. From this point, the Schmitt trigger circuit generates a square shape signal having an amplitude of 3.3 V.

**Figure 7:**
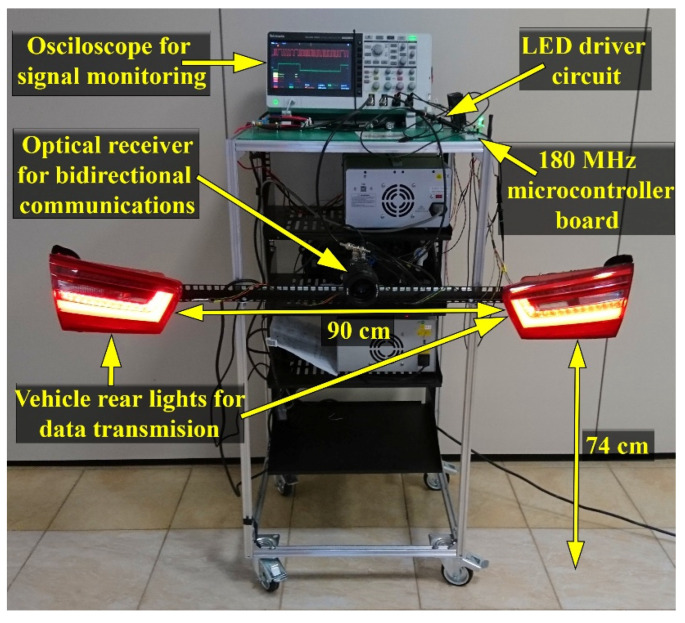
Hardware implementation of the V2V VLC system.

This signal is then fed to the 180 MHz microcontroller board in the final stage, where it is processed in order to extract the information. This process is based on a rising and falling edge identification and pulse width measurement. Complementary to the VLC emitter, the VLC receiver is able to process messages transmitted using DSSS or OOK modulation techniques, SIK, Manchester and Miller encoded messages, having data rates between 3 and 200 kb/s. To fulfill this goal, the microcontroller extracts the required information from the physical header of the data frame. The microcontroller is able to perform the data processing in real-time. Once the information is decoded, the microcontroller determines the BER which is used for performance analysis. Figure 7 shows the hardware implementation of the VLC system, whereas Table 1 and Table 2 present the summary of the VLC system’s parameters.

## 6. Outdoor Testing Procedure

The experimental evaluation of the VLC systems began with the examination of the VLC emitter prototype light radiation pattern. This investigation aimed to establish the similarity between the light radiation pattern of the VLC emitter and the one of commercial vehicles. Additionally, this examination should determine that the proposed VLC prototype is in accordance with existing standards imposed to vehicle lighting systems [66]. The light distribution measurement procedure is similar to the one followed for the determination described in Section 4, whereas the results are presented in Section 7.2.

The second part of the experimental investigation is focused on determining the V2V VLC link performances and the effect of vehicle misalignment on the performances of such VLC applications. To this purpose, this article considers a two-lane scenario where the VLC emitter can be on the same lane or on the adjacent lane with respect to the VLC receiver. Based on this fact, the communication parameters could be influenced in terms of minimum communication distance, BER, and communication range. Therefore, in order to have an adequate assessment concerning the manner in which vehicle misalignment influences these parameters and to move forward from laboratory prototypes to vehicle embedded systems, extensive experimental investigation is required. The projected testing scenario is illustrated in Figure 1.

Similar to the real case scenario, the dynamic situations imposed by the vehicle mobility do not guarantee that the vehicles (i.e., the VLC emitter and the VLC receiver) are in alignment with respect to each other. In order to determine the effect of vehicle misalignment, the V2V system was tested in outdoor conditions, on a two lane road, within the parking lot of the Stefan cel Mare University of Suceava. For this purpose, the VLC emitter (Tx vehicle in Figure 1) was placed on the second lane, whereas the VLC receiver (Rx vehicle in Figure 1) was alternatively placed on the first and on the second lane. The measurement procedure was begun from a one meter V2V longitudinal distance. Next, the V2V distance was gradually increased up to a maximum distance of 75 m.

As the VLC receiver’s field-of-view significantly influences the communication parameters, the VLC prototype has been evaluated for a wide ±53° FOV and a narrower one, of ±20° FOV. For both these cases, the measurements were made in day conditions, under the influence of the sunlight, and in night conditions under the influence of fluorescent light sources that are illuminating the parking lot. For each longitudinal distance and for each scenario, the BER was determined for the VLC receiver alternately placed on each of the two lanes. In order to determine the BER, a predefined message has been cyclically transmitted. The VLC receiver processes the incoming light beam, decodes the data in real-time and computes the BER by matching the received bits with the bits of the original message.

**Figure 8:**
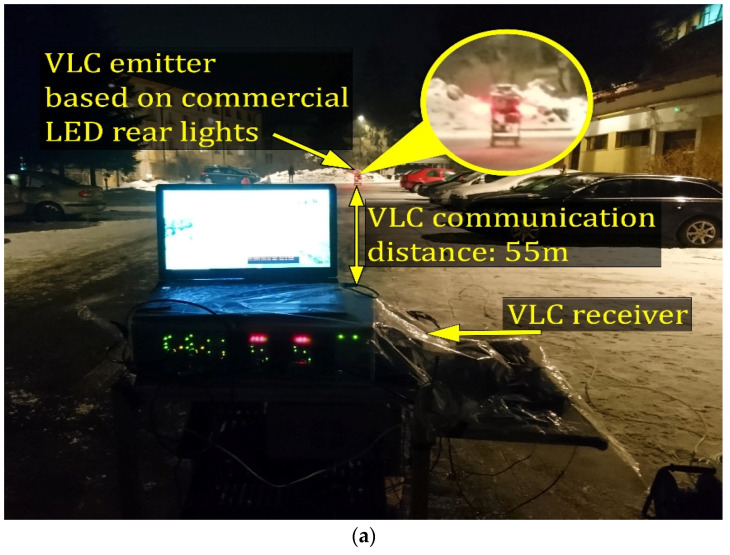

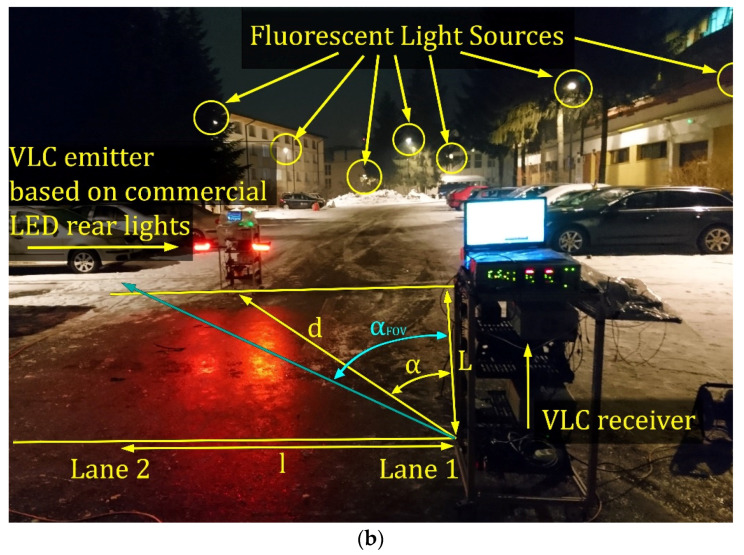
Outdoor experimental testing setup with the VLC emitter and the VLC receiver on the same lane (**a**), and on adjacent lanes (**b**).

In accordance with the specifications of the IEEE 802.15.7 standard, the data were transmitted using OOK modulation, Manchester coding and a 100 kb/s data rate [67]. A summary of the testing parameters is available in Table 3, whereas the experimental testing setup is shown in Figure 8.

## 7. Experimental Results

### 7.1. The Experimental Determination of the Light Radiation for the VLC Emitter

**Figure 9:**
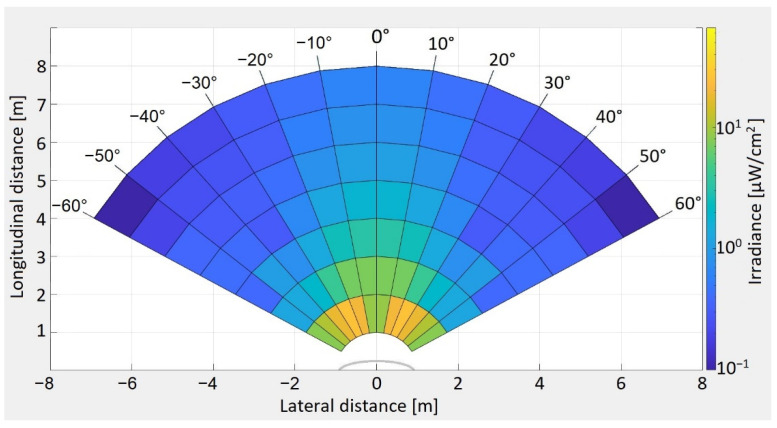
VLC emitter prototype irradiance distribution.

Figure 9 illustrates the VLC emitter light irradiance pattern, presenting the experimentally determined light pattern for a distance between 1 and 8 m, and from 0° to ±60°, the measured values ranging from 26.39 µW/cm^2^ to 0.1 µW/cm^2^. The experimental determination of the VLC emitter light radiation confirms that the developed VLC emitter prototype is in accordance with the vehicle lighting system minimal standards [66], and that it has a similar pattern to the one of existing vehicles. Moreover, if it is to compare the light radiation pattern of the VLC emitter with the ones of vehicle brake lights (Figure 5), one can see that the VLC emitter has a lower optical irradiance. Based on these determinations, one can see that the irradiation pattern of the VLC emitter is very alike with the ones measured for the real vehicles. Therefore, it can be assumed that comparable results could be obtained by a VLC system implemented on real vehicles.

### 7.2. The Experimental Evaluation of the Effect of Vehicle Misalignment and of Inter-Vehicle Distance

**Figure 10:**
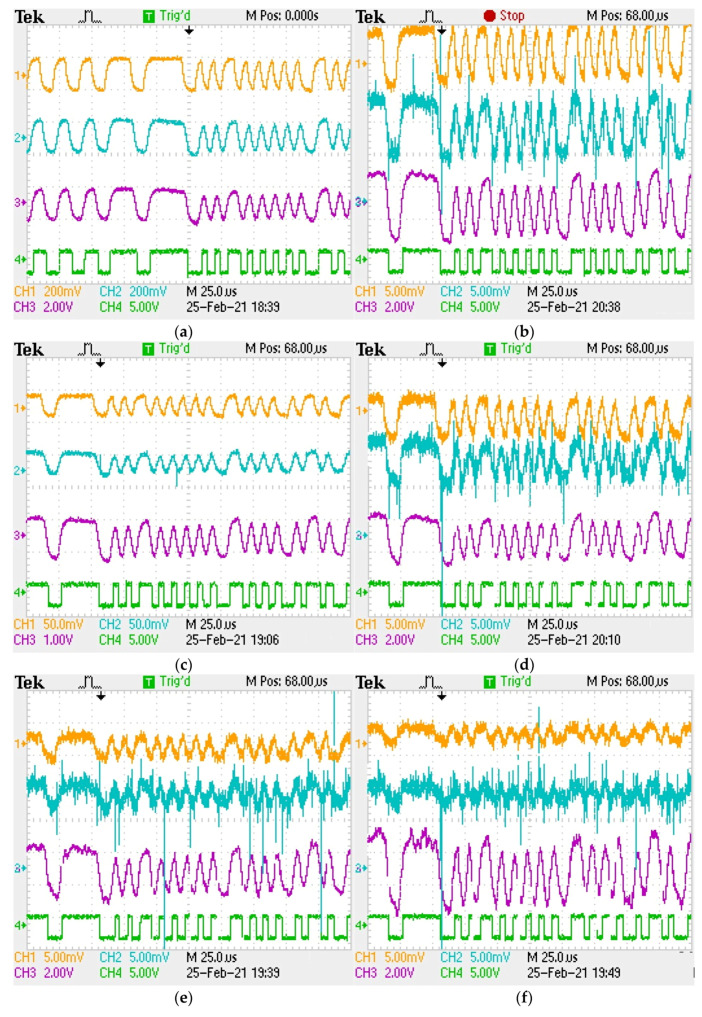
Oscilloscope print screens showing the signal reconstruction process at the level of the different blocks of the VLC receiver: Channel 1 (orange) shows the output of the optical receiver for a 40 dB gain; Channel 2 (cyan) shows the output of the band-pass filter; Channel 3 (purple) shows the output of the amplification block; Channel 4 (green) shows the reconstructed signal which will be used in the data decoding process: (**a**) vehicles on the same lane with 4 m inter-vehicle distance; (**b**) vehicles on adjacent lanes with 4 m inter-vehicle longitudinal distance (close to the minimum communication distance); (**c**) vehicles on the same lane at 10 m distance; (**d**) vehicles on adjacent lanes at 10 m inter-vehicle longitudinal distance; (**e**) vehicles on the same lane at 35 m; and (**f**) vehicles on adjacent lanes at 35 m inter-vehicle longitudinal distance. One can see that as the distance increases, the misalignment effect becomes less stringent.

Once the validation of the VLC emitter confirmed that its irradiation pattern is within the specifications of the existing standards, the next step is to provide an empirical evaluation of the VLC receiver’s ability to reconstruct the data signal and most importantly, to evaluate the effects of vehicle misalignment on the received signal. Therefore, Figure 10 illustrates the signal processing at the VLC receiver level, marking the most important signal reconstruction steps. In order to clearly illustrate the effect of the variable distance and of the vehicle misalignment on the received data signal, these oscilloscope print screens show the measurements taken in night conditions with a limited effect from the outdoor fluorescent lighting system, while using a wide FOV. Thus, Figure 10 focuses on the effect of vehicle misalignment and of the increasing inter-vehicle distance. As one can see, at a short distance (i.e., 4 m in this case), the effect of vehicle misalignment is very stringent, as the amplitude of the front-end stage (i.e., channel 1 signal) is about 20 times lower compared to the case when the vehicles are on the same lane (Figure 10a,b). Although these experiments have been performed in night conditions, the SNR level is low, but the VLC receiver is able to properly reconstruct the data signal. Nevertheless, existing experience indicates that in daytime conditions, the corroboration of vehicle misalignment and of parasitic sunlight could have a highly disruptive effect on the SNR level and in turn, on the overall communication parameters. Going further, as the inter-vehicle distance increases, the incidence angle α at the VLC receiver decreases and the influence of vehicle misalignment is less pronounced. Thus, at 10 m, the amplitudes of the signals differ by about six times (see Figure 10c,d, channel 1), whereas at 35 m, the two signals have approximately the same amplitude (see Figure 10e,f, channel 1).

**Figure 11:**
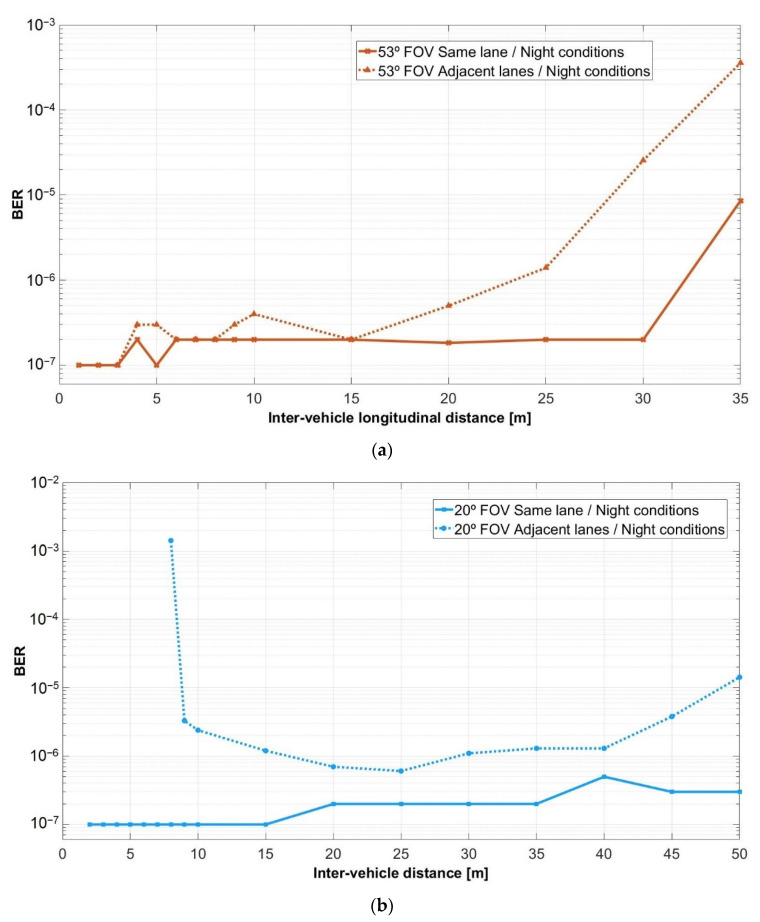
Summary of the V2V experimental BER results for the tests performed in night conditions under the influence of fluorescent lights: (**a**) for a ±53° reception angle; and (**b**) for a ±20° reception angle.

**Figure 12:**
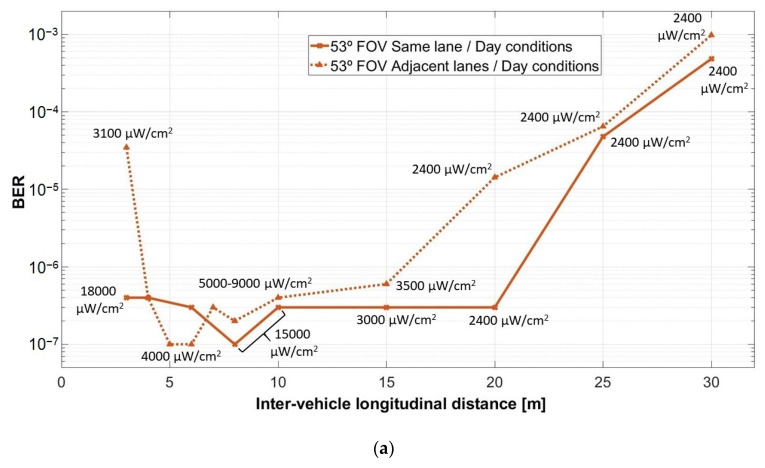

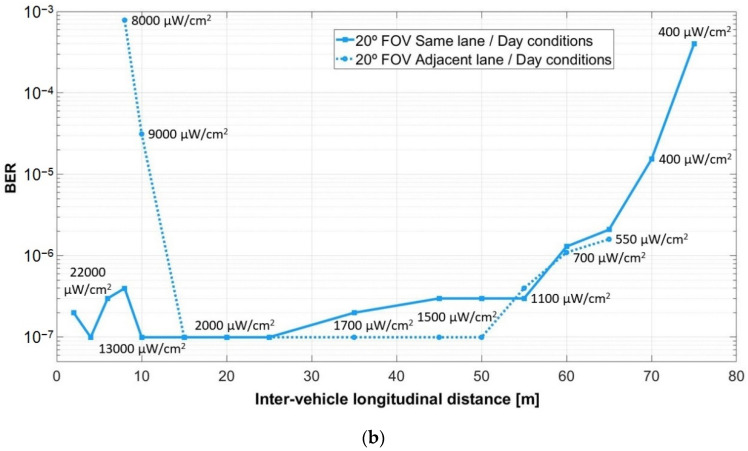
Experimental BER results for the evaluation of the vehicle-to-vehicle VLC link in uncontrolled day conditions: (**a**) for a ±53° reception angle; (**b**) for a ±20° reception angle.

The results of the experimental investigation of the V2V VLC system for the two lane scenario are summarized in Figure 11 and Figure 12. Thus, Figure 11 illustrates the BER results for the experiments performed in night conditions under the influence of the fluorescent lights for wide and for narrow FOV, whereas Figure 12 presents the results of the tests in the same conditions, but during the day time. The experimental BER results clearly show the manner in which the vehicle misalignment influences the performances of V2V VLC links in terms of coverage, minimum reception distance, BER, and communication range. As expected, the effect of vehicle misalignment is stronger at short inter-vehicle longitudinal distances. At short distances (0–2.5 m for a ±53° FOV, respectively 0–8 m for a ±20° FOV), vehicle misalignment generated by the fact that vehicles are located on adjacent lanes, imposes a no-connectivity zone, which is dependent on the VLC receiver FOV. Next to this region (3–5 m for a ±53° FOV, respectively 8–10 m for a ±20° FOV), the wide incidence angle α at the VLC receiver determines an increased BER value of 10^−5^–10^−3^, with respect to the 10^−7^ BER achieved in vehicle-aligned conditions. Further on, the SNR at the VLC receiver located on an adjacent lane with respect to the VLC emitter is still lower (see Figure 10c,d, channel 1), but this SNR difference is compensated by the adequate signal processing. Therefore, the BER remains in the 10^−6^ region. For distances above 15 m, the SNR in both scenarios (i.e., vehicles on the same lane and on adjacent lanes) becomes rather the same, generating similar BER results. As the distance further increases to above 20–25 m for a ±53° FOV and above 50 m for a ±20° FOV, the SNR continues to decrease, generating an abrupt BER increase.

As one can see, fitting the optical collimator, which has a narrower FOV, improves the SNR and extends the 10^−7^–10^−6^ BER region up to 50 m and the maximum communication distance up to 75 m. Thus, these results demonstrate the performances of the VLC technology, which can provide highly reliable V2V links, with BERs that can go as low as 10^−7^ and communication distances up to 75 m. Nevertheless, as one can see, at 75 m the BER significantly increases, reaching a 10^−3^ value. This shows that due to the low optical power, this is the range limit of the current configuration. Although in some applications the 10^−3^ BER value is adequate, in communication-based vehicle safety applications, this BER limit is rather high. On the other hand, these results have been achieved without the usage of forward error correcting protocols. However, communication-based vehicle applications will integrate FEC codes, which will have the ability to improve the BER performances [67].

The 75 m communication limit is determined by three main causes. First of all, the VLC emitter limited the optical power, which is determined by the optical system main lighting function. In order to be in accordance with the lighting regulations and the necessity to avoid glaring other drivers, the optical power of a lighting device cannot be increased above a certain limit. Nevertheless, there is still room to improve the efficiency of the lighting system, so that a better range can be achieved in the future. Another aspect that determines the communication range is given by the VLC receiver sensibility, which is in turn determined by the photosensitive element responsivity (A/W) and by the signal-collecting area. As the proposed VLC receiver uses a highly sensitive optical receiver and a 2-inch optical lens, it is rather difficult to further improve these aspects. From a different point of view, the communication distance could be slightly enhanced by reducing the optical clock frequency and in turn the data rate. These measures would enable a higher gain at the transimpedance circuit level. Last but not least, the communication range could be further increased by enhancing the VLC receiver ability to process low SNR signals. From this point of view, as the performances in terms of low SNR signal processing algorithms will be further improved, the communication distance of such systems will be improved as well.

The results of the experimental investigation performed in night conditions show that the VLC receiver is able to maintain the connectivity, even under exposure to the fluorescent lighting system. Furthermore, the results of the day tests have shown that the usage of an optical filter together with a proper signal processing algorithm enable the VLC receiver to work in the presence of strong sunlight interferences that can go up to 22,000 µW/cm^2^. In such conditions, with a proper VLC system, one can conclude that the influence of the sunlight can have a limited impact over the BER variations, compared to the influence of vehicle misalignment.

The experimental results also showed that the performances of the link were slightly better in day conditions, rather than in night conditions, as would be expected. Similar to a real case scenario in which roads are illuminated at night, the night experiments were conducted in a well-illuminated parking lot (please see Figure 8b). Thus, the lower BER achieved during these tests is a consequence of the disruptive effect generated by the fluorescent light sources. It should be remembered here that the daylight introduces a strong but easy-to-filter DC component, whereas the fluorescent lights introduce a modulated 100 Hz signal, which is slightly more difficult to completely eliminate. It should be also mentioned that when the VLC receiver uses a rather narrow band-pass optical filter centered on the red wavelength, the effect of sunlight is significantly mitigated, which in turn generates a lower BER compared to a standard day scenario. A similar situation has been found in [22]. In this case, the experimental results also showed that the influence of the fluorescent lights is higher than the one of natural light.

## 8. Discussions on the Results, Contributions of this Work, and Perspectives

One of the most important aspects that emerges based on the experimental results analysis is related to the opportunity of using the VLC technology in V2V applications. As the results show, BERs as low as 10^−7^ can be achieved for relatively long distances, even without the use of forward error correcting protocols, whereas the use of such techniques can further improve these results. In terms of communication range, the experiments demonstrated a maximum communication distance of 75 m. As far as we know, this is the longest V2V VLC link established based on a set of commercial vehicle rear lights. Moreover, this communication distance could be further increased for the case of emergency brake applications as the irradiance distribution of vehicle brake lights is significantly higher than the one of the VLC emitter used for these tests. In comparison, the V2V VLC system evaluated in [36], provided a communication distance of less than 20 m, whereas the system from [44] can deliver ranges up to 45 m. It should be pointed out here that the current research focused on automotive VLC systems is mainly orientated toward traffic Infrastructure-to-Vehicle (I2V) connections, as the VLC emitters developed based on transportation infrastructure elements (i.e., traffic lights, street lighting systems) have a significantly higher optical power, enabling longer communication distances. Even so, there are only few works [30,31] that report I2V-related VLC results with communication ranges above 75 m, whereas most of the existing systems provide communication distances of up to 50 m [21,22,23,24,26,27,28,29]. In this context, the 75 m communication range reported in this work provides important evidence that, step by step, the performances of automotive VLC systems are improving.

Another very important aspect that results from the experimental tests is related to the fact that the VLC technology is able to provide V2V connectivity, even in vehicle misalignment conditions. Although the issues related to the effect of vehicle misalignment on V2V VLC performances have been theoretically analyzed in other works [49,50,57,58], this is the first work that provides a consistent experimental evaluation of such a scenario, while providing a step by step comparison with the case when the vehicles are aligned. The authors of [44] provide a highly valuable experimental demonstration of a V2V VLC link in mobile conditions, and identify the distance and the incidence angle as the two main problems affecting the performances of such connections. Nevertheless, the authors of [44] do not provide a comprehensive analysis of the vehicle misalignment problem.

In this context, this article shows that: (i) vehicle misalignment influences the performances of the V2V link in terms of minimum communication distances, which are determined by the VLC receiver’s FOV; this leads to an initial zone where there is no connectivity, followed by a region where the BER is significantly higher compared to the case when the vehicles are aligned; (ii) optical lens, optical filters and adequate signal processing reduce the effect of vehicle misalignment for distances above the minimum distance, enabling the VLC system to provide BERs of the same order for both situations (i.e., vehicles on the same lane, or on adjacent lanes). To do so, the VLC receiver must be able to actively compensate the fact that the received optical power is decreasing, affecting in turn the SNR; (iii) as the longitudinal inter-vehicle distance is increasing, the incidence angle α at the VLC receiver decreases and the effect of vehicle misalignment is mitigated. From this point, the performances of the V2V VLC link will predominantly be affected by the increasing distance.

The experimental results also reconfirmed the importance of an adequate optical collecting front-end component showing the benefits of optical lens and of optical filters. The optical lenses increase the light-collecting region, whereas the optical filters only allow the passage of the wavelengths of interest. Therefore, both solutions contribute to an important SNR enhancement. Moreover, the usage of a narrow FOV further improves the SNR, as it eliminates part of the light coming from the sides but at the expense of an increased initial no-connection zone. Based on these techniques, one can see that the BER performances have been improved by up to four orders of magnitude, as it can be seen in Figure 12a,b for distances over 25 m. Moreover, the optical lens and the narrower FOV have increased the communication distance from 35 m to 75 m. On the down side, the narrow FOV makes it difficult for the VLC receiver to establish the LoS with the VLC emitter, affecting, in turn, the mobility. However, several solutions to this problem emerge. Cooperative and relay-assisted protocols are an example of such solutions where a VLC receiver that is not within the LoS with the VLC emitter receives the information with the help of an intermediate node. The efficacy of this method has been intensively investigated in indoor VLC applications [18,19]. This solution has also been found to be suitable in inter-vehicle applications and the results showed that the connectivity on curved roads can be improved with the help of a relay vehicle [58]. Experimental demonstrations of the usage of relay-assisted VLC in vehicular applications are available in [68,69]. In such cases, it has been experimentally demonstrated that a VLC link can be established with a VLC receiver that is not within the LoS by using an intermediate VLC node.

The effect of vehicle misalignment could be also mitigated based on the integration of context and environment-adaptive techniques [47,48]. In this case, an adaptable FOV can be used [41]. In [22], it has been demonstrated that the VLC receiver reception angle can be increased with the help of a proper optical lens. All these works prove that the research community is able to come up with solutions that will enable the use of the VLC technology in communication-based vehicle safety applications.

Last but not least, it should be remembered that in addition to vehicle misalignment, the SNR at the VLC receiver level can also be influenced by weather phenomena that influences light passage (i.e., fog, rain, snowfall). Nevertheless, the research community has come up with solutions to reduce these effects. According to [70], snowfall is the most disruptive weather phenomena for VLC applications, reducing the communication distance by 20–80%. The effect of snowfall and of blizzards on vehicular VLC performances has been experimentally evaluated in [23,24]. The results showed that even if the BER can be affected, the VLC link can be maintained as active, even in such unfriendly conditions. The solutions to such problems are generally based on an optical lens, improved signal processing techniques, which enable data recovery from low SNR signals, and FEC protocols to improve BER results in low SNR conditions. Therefore, as these issues have been already addressed by the research community, these aspects are only briefly mentioned in this manuscript.

In these circumstances, one can see that the performances of automotive VLC systems are constantly improving. Therefore, the results of this article provide new evidence concerning the benefits associated with the use of the VLC technology in vehicular applications. Thus, in addition to an envisioned fast spreading possibility and a low cost generated by the wide distribution of the LED light sources, VLC technology can provide decent communication distances and a high reliability.

## 9. Conclusions

As interest in new solutions for improved traffic safety is increasing, the use of communication-based vehicle safety applications appears to be highly promising. In this context, this article addressed some of the issues associated with the exploitation of the VLC technology in V2V applications, focusing on an experimental-centered approach. To this goal, a vehicle rear-lighting-based VLC emitter was designed, implemented and tested, together with a VLC receiver, in order to attain the best possible results for this scenario. The V2V VLC prototype has been intensively tested in outdoor surroundings, in night conditions under the exposure of fluorescent lights and in daylight conditions as well. The experimental tests have been focused on two main concerns: (i) determining the maximum communication distances attainable with the proposed prototype and (ii) analyzing the effect of vehicle misalignment. The experimental results demonstrated a record communication range of 75 m with a relatively low-power VLC emitter based on vehicle rear lights, with BERs as low as 10^−7^. It has been also demonstrated that with the proposed signal processing plan, the VLC receiver is able to compensate the SNR decrease, maintaining a decent BER for an extended range. This article has also reconfirmed the benefits of an adequate optical collecting system. Thus, optical filters and a narrow FOV obtained with optical lenses can significantly optimize a VLC receiver. Based on these results, this article has provided important evidence concerning the ability of VLC technology to establish reliable V2V links.

Future work on this topic will be orientated toward the evaluation of the VLC system in real driving situations. Nevertheless, these experiments are highly complex in terms of connectivity, safety and measurement repeatability. In order to achieve this goal, a test track for intelligent cars is under construction as a necessary step for future developments.

## Figures and Tables

**Table 1:** Summary of the VLC emitter parameters.

Parameter	Feature/Values
Optical source	Commercial vehicle LED rear lights
Emitted Irradiance	No stronger than the one of existing commercial vehicles (see details in Figure 5 and in Section 7.1)
VLC emitter center wavelength	620 nm
Available modulation techniques	DSSS, OOK
Available coding techniques	SIK, Manchester, Miller
Available data rates	3–200 kb/s
VLC emitter height	74 cm (similar to a real vehicle)
Distance between the two stoplights	90 cm (similar to a real vehicle)

**Table 2:** Summary of the VLC receiver parameters.

VLC Receiver Stage	Parameter	Feature/Values
Front-end	VLC receiver FOV	±53° respectively ±20°
	VLC optical receiver	PDA100A optical detector with adjustable gain
	VLC receiver optical filter center wavelength	645 ± 40 nm
Signal conditioning	Amplification	Up to 5000 gain, with automatic gain control
	Signal filtering	- 1 kHz high-pass 2nd order Bessel filter - adjustable 4th order Bessel low-pass cut-off frequency filter (200 kHz for these tests)
	Square signal reconstruction	Based on Schmitt trigger circuit
Data processing	Hardware	Microcontroller board based on an ARM Cortex M4 180 MHz processor
	Data processing	Based on rising and falling edge identification and pulse width measurement
	Data decoding	Real-time data extraction for data modulated using DSSS and OOK modulation, SIK, Manchester and Miller coding, with data rates between 3–200 kb/s
	Monitored parameters	Real-time BER computing without forward error correcting codes
Other VLC receiver parameters	Height of the optical detector	74 cm

**Table 3:** Summary of the experimental parameters.

Parameter	Feature/Values
Testing conditions	Outdoor, uncontrolled conditions
Testing scenario	Two lane V2V communication with: vehicles on the same lane vehicles on adjacent lanes
Lighting conditions	- Day conditions with variable irradiance of sunlight between 400–22,000 µW/cm^2^ - Night conditions under the influence of fluorescent lights
VLC emitter	LED-based vehicle rear lights
VLC emitter height	74 cm (i.e., similar as commercial vehicles)
Distance between the two vehicle rear lights	90 cm (i.e., similar to the case of a commercial vehicle)
Width of a lane	3.5 m
Emitter–Receiver (V2V) distance	1–75 m
Emitter–Receiver (V2V) angle	0°–49°
VLC receiver	Photodiode-based
VLC receiver height	74 cm
Modulation technique	OOK
Coding technique	Manchester
Data rate	100 kb/s
Measured parameter	Real-time BER determination without the usage of forward error correcting protocols
